# Epidermal Growth Factor Receptor Status in Circulating Tumor Cells as a Predictive Biomarker of Sensitivity in Castration-Resistant Prostate Cancer Patients Treated with Docetaxel Chemotherapy

**DOI:** 10.3390/ijms17122008

**Published:** 2016-11-30

**Authors:** Takatsugu Okegawa, Naoshi Itaya, Hidehiko Hara, Mitsuhiro Tambo, Kikuo Nutahara

**Affiliations:** Department of Urology, The University of Kyorin, 6-20-2 Shinkawa, Mitaka, Tokyo 181-8611, Japan; nao@ks.kyorin-u.ac.jp (N.I.); sdjdx005@yahoo.co.jp (H.H.); tanbodes@ks.kyorin-u.ac.jp (M.T.); kinuta@ks.kyorin-u.ac.jp (K.N.)

**Keywords:** circulating tumor cells, castration-resistant prostate cancer, biomarker, EGFR

## Abstract

Objective: We examined whether epidermal growth factor receptor (EGFR) expression in circulating tumor cells (CTCs) can be used to predict survival in a population of bone-metastatic castration-resistant prostate cancer (mCRPC) patients treated with docetaxel chemotherapy. Methods: All patients with mCRPC who had experienced treatment failure with androgen-deprivation therapy and had received docetaxel chemotherapy were eligible. CTCs and EGFR expression in CTCs were enumerated with the CellSearch System in whole blood. This system is a semi-automated system that detects and enriches epithelial cells from whole blood using an EpCAM antibody-based immunomagnetic capture. In addition, the EGFR-positive CTCs were assessed using CellSearch^®^ Tumor Phenotyping Reagent EGFR. Results: The median CTC count at baseline before starting trial treatment was eight CTCs per 7.5 mL of blood (range 0–184). There were 37 patients (61.7%) who had ≥5 CTCs, with median overall survival of 11.5 months compared with 20.0 months for 23 patients (38.3%) with <5 CTCs (*p* < 0.001). A total of 15 patients (40.5%, 15/37) with five or more CTCs were subjected to automated immunofluorescence staining and cell sorting for EGFR protein. Patients with EGFR-positive CTCs had a shorter overall survival (OS) (5.5 months) than patients with EGFR-negative CTCs (20.0 months). CTCs, EGFR-positive CTCs, and alkaline phosphatase (ALP) were independent predictors of overall survival time (*p* = 0.002, *p* < 0.001, and *p* = 0.017, respectively). Conclusion: CTCs may be an independent predictor of OS in CRPC treated with docetaxel chemotherapy. The EGFR expression detected in CTCs was important for assessing the response to chemotherapy and predicting disease outcome.

## 1. Introduction

Castration-resistant prostate cancer (CRPC) is a complex process involving many different signal transduction pathways. The epidermal growth factor receptor (EGFR) has been reported to provide an important mechanism for the progression of CRPC [1,2]. Hyperactivity of EGFR is related to androgen independence of prostate cancer cells. Levels of EGFR immunoreactivity were increased in hormone-independent human prostate cancer cell lines [3,4]. Several investigators demonstrated EGFR expression as high as 90%–100% in tissue from patients with metastatic CRPC [5,6].

Several groups have reported that the number and characteristics of circulating tumor cells (CTCs) in cancer patients are parallel to tumor progression and response to treatment [7,8,9]. CTCs are generally thought to separate from tumors of patients with advanced cancer prior to detection in circulation. The developed CellSearch System (Veridex) was designed to detect CTCs in whole blood. This system was developed using epithelial cell adhesion molecule (EpCAM) and cytokeratin antibody-based immunomagnetic capture and automated staining methodology. With this system, highly reproducible quantitative results from different laboratories can be obtained. Isolation and capture techniques of CTCs have been reported by several groups; however, only CellSearch has been analytically validated and approved by the Food and Drug Administration (FDA) [7,9]. Primary studies established that CTCs can be used in conjunction with other modalities for monitoring patients with different metastatic cancers [8,9]. Recent studies have demonstrated that CTC markers may change over the course of therapy [10,11,12,13].

We therefore examined the surface EGFR expression levels in the prognostic and therapeutic value of CTCs before docetaxel chemotherapy in a population of bone-metastatic castration-resistant prostate cancer (mCRPC) patients at Kyorin University.

## 2. Results

### 2.1. CTC Count

The median CTC count at baseline before starting the trial treatment was 8 CTC per 7.5 mL of blood (range 0–184). Overall, 23 patients (38.3%) had a CTC count of <5, while 37 patients (61.7%) had a CTC count of ≥5.

### 2.2. Baseline CTC Count Correlation with Patient Characteristics

The correlation of CTC count distribution and baseline characteristics is shown in Table 1. Multivariate analysis demonstrated that higher CTC counts were associated with: ALP > UNL (*p* = 0.002), hemoglobin level of <11.5 g/dL (*p* = 0.034), PSA of >30 ng/mL (*p* = 0.042) EOD > 3 (*p* = 0.003), and a Gleason score >9 (*p* = 0.021). Patients with bone plus lymph node metastases had a higher median CTC count than patients with only bone metastases (*p* = 0.014).

### 2.3. Analysis of Epidermal Growth Factor Receptor (EGFR) Protein Expression in CTCs

**Figure 1:**
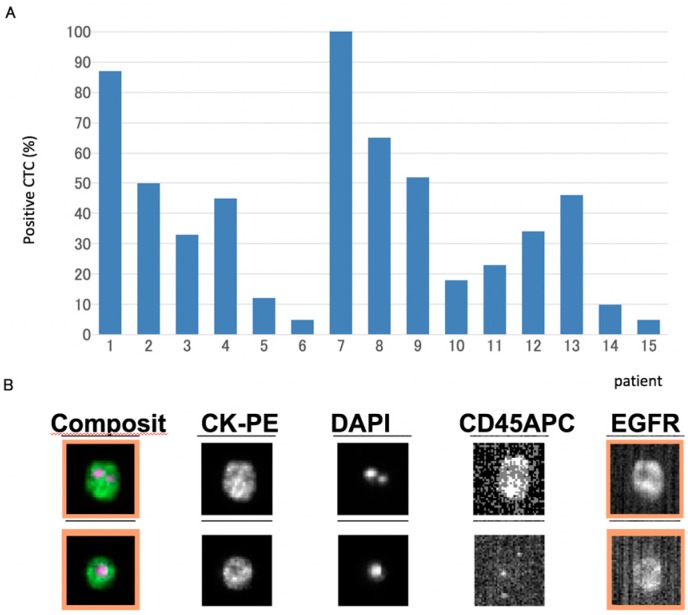
(**A**) Quantitation of epidermal growth factor receptor (EGFR) expression in CTCs by automated immunofluorescence assay. The percentage of EGFR-positive CTCs relative to total CTCs was analyzed in samples with >5 CTCs and (**B**) A patient sample shows an individual CTC stained positive for EGFR expression.

There were 15 patients (40.5%, 15/37) with five or more CTCs subjected to automated immunofluorescence staining and cell sorting for EGFR protein. The percentage of CTCs positive for EGFR ranged from 5% to 100%, with a median of 39%. The distribution of percentages is shown in Figure 1.

### 2.4. Multivariate Analyses Indicate that CTCs at Baseline Are an Independent Predictor of Overall Survival

**Figure 2:**
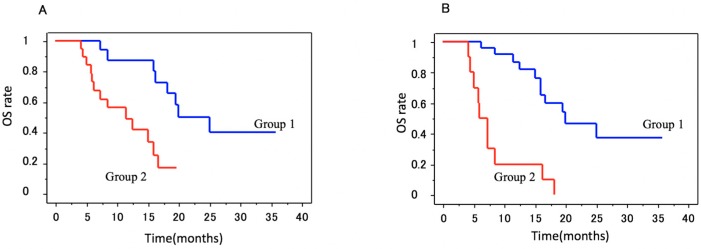
(**A**) Kaplan—Meier analysis of baseline CTCs to predict overall survival time in patients with mCRPC. The overall survival time was significantly shorter in patients with ≥5 CTCs. A total of 23 patients (38.3%) with <5 CTCs/7.5 mL of blood (group 1) had a median overall survival time of 20.0 months compared with 11.5 months in 37 patients (61.7%) with ≥5 CTCs (group 2) (*p* = 0.0017, log-rank test) and (**B**) Kaplan—Meier analysis of baseline CTC-EGFR to predict overall survival time in patients with mCRPC. The overall survival time was significantly shorter in patients with EGFR-positive CTCs. There were 45 (75.0%) with EGFR-negative CTCs (group 1) that had a median overall survival time of 20.0 months compared with 5.5 months in 15 patients (25.0%) with EGFR-positive CTCs (group 2) (*p* < 0.001, log-rank test).

The survival rates were calculated from the time of the baseline blood sampling. The patient charts were examined to determine OS, which ranged from 5.0 to 37.0 months (mean 13.7 ± 9.2, median 15.3). Multivariate analysis demonstrated that patients with a CTC count of ≥5 at baseline had a shorter OS (11.5 months) than patients with a CTC count of <5 (20.0 months) (Figure 2A). Multivariate analysis demonstrated that patients with EGFR-positive CTCs had a shorter OS (5.5 months) than patients with EGFR-negative CTCs (20.0 months) (Figure 2B). CTC count of ≥5, EGFR-positive CTCs, and ALP > UNL were independently correlated with a poor OS (Table 2).

## 3. Discussion

Recently, CTCs have been widely recognized as a prognostic biomarker for prostate cancer patients using the FDA-cleared CellSearch System. Numerous studies have demonstrated the association between CTC baseline levels and clinical outcomes in metastatic patients. Scher et al. have reported that the combination of CTC number and LDH level was a surrogate for survival at the individual-patient level in the COU-AA-301 trial comparing abiraterone acetate plus prednisone versus prednisone alone for patients with metastatic CRPC [13].

We found CTCs in 61.7% of CRPC patients with pre-docetaxel using a cutoff of five cells per 7.5 mL of blood. A threshold of ≥5 CTCs per 7.5 mL of blood was used to evaluate the suitability of CTCs to predict survival using FDA-cleared this system. We examined the usefulness of CTCs for predicting survival in 60 CRPC patients treated with docetaxel chemotherapy. Patients with <5 CTCs per 7.5 mL of blood had a median OS time of 20.0 months compared with 11.5 months in patients with ≥5 CTCs (*p* < 0.001). The results demonstrated that the evaluation of CTC levels accurately and reproducibly predicted clinical outcome, as previously reported [14]. Apart from a CTC count of ≥5, ALP > UNL was also independently correlated with a poor OS. Goldkorn et al. reported the correlation with CTCs by analyzing CTCs in 161 patients with CRPC treated with first-line TXT-based therapy in SWOG S0421 [12]. The authors demonstrated that baseline CTC counts were correlated with recognized prognostic markers, including PSA, alkaline phosphatase, hemoglobin, liver disease, and bone pain. Median OS was 26 months for the group of CTCs of <5 versus 13 months for those patients with CTCs ≥5 per 7.5 mL at baseline. Unfortunately, the relationship with LDH was not assessed, as in this study. Taken together, CTCs at baseline are a strong, independent prognostic biomarker pre-docetaxel.

We identified the surface EGFR expression in CTCs before docetaxel chemotherapy in the CRPC. EGFR plays an important role in cell proliferation, migration, motility, invasion, and survival in malignant cells. EGFR is often overexpressed and is associated with aberrant signaling leading to aggressive malignancies and poor patient survival rate [15]. It has been reported that nearly 30% of PCa cases overexpress EGFR and that deregulation of EGFR-mediated signaling pathways is associated with poor clinical outcomes [16,17]. Theil et al. detected tumor-associated transcripts of EGFR in patients with metastatic PCa in 42.8% using CellCollector [18]. Shaffer et al. detected EGFR expression in 18/20 (90%) of CTCs in patients with metastatic PCa using the CellSearch system [19]. In other cancers, EGFR expression in CTCs is associated with poor prognosis and overall survival. Vila et al. reported a noticeable positive correlation between the number of EGFR-positive CTCs and worse prognosis, and overall survival in prostate cancer, breast cancer and lung cancer [20]. Serrano et al. reported that EGFR-positive CTCs indicate vulnerability to visceral disease [21]. Among our analyzed CRPC blood samples with a CTC count of ≥5 (*n* = 37), 40.5% (*n* = 15) were positive for EGFR in CTCs. Patients with EGFR-positive CTCs had a shorter OS (5.5 months) than patients with EGFR-negative CTCs (20.0 months). The tumor cells isolated from the blood may provide a better overall reflection of the biological heterogeneity of the disease than tumors from a specific site. Shaffer et al. reported that the percentage of EGFR-positive CTCs in the 20 samples analyzed ranged from 0% to 100%, as in this study [19]. These results suggest progression of cancer and may be a valuable tool in the near future. We need to characterize CTCs in PCa as well as the overall number of CTCs in order to gain a better understanding.

Several groups reported the clinical importance of androgen receptor in CTCs as a marker. Androgen receptor splice variant 7 (AR-V7), the most commonly expressed AR-V in CTCs, has been shown to provide a mechanism for development and progression of CRPC. Antonarakis et al. reported AR-V7-positive patients were associated with resistance to treatment with abiraterone and enzalutamide [22]. In addition, AR-V7 in CTCs from patients with metastatic CRPC is not associated with primary resistance to docetaxel chemotherapy. In AR-V7-positive patients, docetaxel chemotherapy appears to be more efficacious than enzalutamide or abiraterone therapy [23,24]. Analyses of tumor heterogeneity, as in this study, are very important for identifying which CTCs in CRPC have aggressive or dormant behaviors.

## 4. Materials and Methods

### 4.1. Patient Characteristics

The clinical characteristics of the patients are shown in Table 3. There were 60 mCRPC patients treated at Kyorin University Hospital between April 2012 and March 2014 who were enrolled. All patients with mCRPC who had experienced treatment failure due to androgen-deprivation therapy and had received docetaxel-based chemotherapy were eligible. All patients received 4 mg zoledronic acid every four weeks in addition to androgen-deprivation therapy. Disease progression was defined as documented PSA progression according to the Prostate-Specific Antigen Working Group 2 criteria and a PSA of >5 ng/mL, or as objective progression by Response Evaluation Criteria in Solid Tumors (RECIST) criteria for patients with measurable disease. The ethics committee of the university approved the study protocol according to the Declaration of Helsinki (number: H26-005-03 and 31-05-2014). All patients provided written consent.

### 4.2. Drug Administration

Docetaxel (70–75 mg/m^2^) and dexamethasone (8 mg/body) were given by intravenous infusion every three to four weeks. Subjects were simultaneously treated with hormonal therapy with an luteinizing hormone-releasing hormone analogue and daily oral dexamethasone (0.5–1.0 mg/day).

There were 28 (44%) patients who had received cabazitaxel after they were treated with docetaxel. Cabazitaxel was given at a dose of 25 mg/m^2^ intravenously every three to four weeks. A total of 32 (56%) patients received best care support.

### 4.3. Samples

Blood samples of patients diagnosed with mCRPC and treated with docetaxel chemotherapy were drawn into CellSave^®^ Preservative Tubes (Immunicon, Huntingdon Valley, PA, USA) or an ethylene-diamine-tetra-acetic acid (EDTA) Vacutainer^®^, an evacuated blood drawtube containing EDTA as an anticoagulant and a cellular preservative. All samples were maintained at ambient temperature, with those in EDTA tubes processed within 6 h of collection and those in CellSave Preservative Tubes processed within 72 h of collection.

### 4.4. Isolation and Enumeration of Circulating Tumor Cells (CTCs—CellSearch System)

The FDA-cleared CellSearch System (Veridex LLC, Warren, NJ, USA) was used for the isolation and enumeration of CTCs. This system was described previously [25]. In brief, 10 mL samples of blood were drawn into a CellSave Preservative Tube. The Cell Search Epithelial Cell Kit (Veridex LLC, Warren, NJ, USA) consists of EpCAM antibody-covered ferroparticles, and processed on a CellTracks Autoprep (Immunicon). Enriched epithelial cells were identified by immunofluoresence staining with Cell Track Analyzer II (Immunicon). Cells were scored as CTCs when 4’ 6-diamidino-2-phenylindole (DAPI)-stained nucleated cells expressed cytokeratin 8, 18 and 19, excluding White Blood Cell contamination by negative selection with CD-45 staining. Automatically selected images were reviewed by the operator for identification. In addition, the EGFR-positive CTCs were assessed using CellSearch^®^ Tumor Phenotyping Reagent EGFR [19]. The results of CTCs and EGFR expression in the CTCs are always expressed as the number of cells per 7.5 mL of blood. We identified the surface EGFR expression in CTCs before docetaxel chemotherapy in the CRPC.

### 4.5. Statistical Analysis

The time to death was defined as the elapsed time between the date on which blood was drawn and the date of death or last follow-up. Wilcoxon’s rank sum test or Fisher’s exact test was used to test for significant differences in the proportion of patients with CTCs greater than a particular threshold among the various patient characteristics. A threshold of ≥5 CTCs per 7.5 mL, which has been shown to be prognostic in a number of prostate cancer trials, was used for overall survival (OS) analysis. Patients with five or more CTCs were subjected to automated immunofluorescence staining and cell sorting for EGFR protein.

The median survival of patients with values greater than or equal to several PSA thresholds was evaluated to establish a PSA threshold (cut-off: 30 ng/mL) to stratify the patients into two groups. The extent of bone metastasis was classified by the extent of disease (EOD) grade according to the method of Soloway et al. [26]. The criteria for anemia and development of bone metastases were modified to hemoglobin (Hb) of <11.5 g/dL and alkaline phosphatase (ALP) above the upper normal limit (UNL) at our hospital. Median OS was determined for patients with ≥5 CTCs per 7.5 mL of blood at baseline and specified intervals. The patient charts were examined retrospectively to determine the OS time. The correlation of CTCs with OS on Kaplan-Meier survival curves was examined using the log-rank test. Cox logistic regression analysis was performed with nine categorical variables: PSA, Gleason score, EOD, Hb, ALP, lactate dehydrogenase (LDH), albumin, and CTCs.

## 5. Conclusions

The clinical significance of circulating tumor cell (CTC) detection has been demonstrated in pre-docetaxel chemotherapy, but further study is needed for the molecular characterization of CTCs. Epidermal growth factor receptor (EGFR) expression in CTCs is associated with poor clinical outcomes. The analysis of CTCs has the potential to become one of the most promising tests in oncology.

## Figures and Tables

**Table 1:** Association between circulating tumor cells (CTC) and baseline characteristics.

Variables	N	CTC/7.5 mL		*p*-Value
		Mean	Range	
CTC count at base	60	8	0–184	0.042
PSA (ng/mL)				
<30	30	2	0–3	0.021
≥30	30	15	0–184	
Biopsy Gleason score				
7–8	29	2	0–6	0.003
9–10	31	19	0–184	
EOD				
1–2	39	1	0–14	0.034
3–4	21	23	0–184	
Hemoglobin (g/dL)				
<11.5	35	18	0–184	0.059
≥11.5	25	5	0–13	
Serum albumin (g/dL)				
<3.7	28	19	0–184	0.059
≥3.7	22	4	0–12	
Alkaline phosphatase (IU/L)				
<313	21	3	0–34	0.002
≥313	39	18	0–184	
Lactate dehydrogenase (IU/L)				
<226	26	2	0–18	0.066
≥226	34	19	0–184	
Disease involvement				
Only bone	42	6	0–45	0.014
Bone plus node	18	15	0–184	

CTC median and range are expressed as cells per 7.5 mL of blood. The extent of bone metastasis was classified by the extent of disease (EOD) grade.

**Table 2:** Baseline prognosis factors for overall survival.

Variables	No.	Univariate Analysis	Multivariate Analysis	Hazard Ratio	95% CI
CTC count					
CTC < 5	23	0.001	0.002	3.24	1.2–4.3
CTC ≥ 5	37				
CTC-EGFR					
(−)	45	<0.001	<0.001	4.01	1.1–6.8
(+)	15				
PSA (ng/mL)					
<30	30	0.015	0.053	4.01	1.1–6.8
≥30	30				
Biopsy Gleason score					
7–8	29	0.011	0.42	1.02	0.6–2.9
9–10	31				
EOD					
1–2	39	0.012	0.285	1.49	0.5–6.8
3–4	21				
Hemoglobin (g/dL)					
<11.5	35	0.028	0.247	1.11	0.6–5.6
≥11.5	25				
Serum albumin (g/dL)					
<3.7	38	0.029	0.312	1.08	0.7–5.9
≥3.7	22				
Alkaline phosphatase (IU/L)					
<313	21	0.003	0.017	2.46	1.2-5.3
≥313	39				
Lactate dehydrogenase (IU/L)					
<226	26	0.008	0.121	1.9	0.5–2.9
≥226	34				
Disease involvement					
Only bone	42	0.071	0.226	1.17	0.6–2.4
Bone plus node	18				

The extent of bone metastasis was classified by the extent of disease (EOD) grade.

**Table 3:** Patients’ clinical characteristics per study group.

No. of Patients	*n* = 60
Mean age	71 (57–82)
median PSA (ng/mL)	33.7 (9.4–3241.7)
≤10.0	2 (3.3%)
10.1–20	12 (20.0%)
20.1–30	16 (26.7%)
30.1–40	7 (11.7%)
40.1–50	7 (11.7%)
50.1–100	11 (18.3%)
≥100.1	5 (8.3%)
Gleason score	
7	8 (13.3%)
8	21 (35.0%)
9	19 (31.7%)
10	12 (20.0%)
EOD	
1	20 (33.3%)
2	19 (31.7%)
3	13 (21.7%)
4	8 (13.3%)
Disease involvement	
Only bone	32 (53.3%)
Bone plus node	28 (46.7%)
WHO perfomance score	
0	56 (93.3%)
1	4 (6.7%)
Median Hemoglobin (g/dL)	10.6 (9.1–12.9)
Median serum albumin (g/dL)	3.4 (2.9–4.1)
Median alkaline phosphatase (IU/L)	342 (178–561)
Median lactate dehydrogenase (IU/L)	265 (189–438)

Number (and percentage) of patients shown unless otherwise indicated. The extent of bone metastasis was classified by the extent of disease (EOD).
